# Use of SSRI and SNRI Antidepressants during Pregnancy: A Population-Based Study from Denmark, Iceland, Norway and Sweden

**DOI:** 10.1371/journal.pone.0144474

**Published:** 2015-12-14

**Authors:** Helga Zoega, Helle Kieler, Mette Nørgaard, Kari Furu, Unnur Valdimarsdottir, Lena Brandt, Bengt Haglund

**Affiliations:** 1 Centre of Public Health Sciences, Faculty of Medicine, University of Iceland, Reykjavik, Iceland; 2 Centre for Pharmacoepidemiology, Department of Medicine, Solna Karolinska Institutet, Stockholm, Sweden; 3 Department of Clinical Epidemiology, Institute of Clinical Medicine, Aarhus University Hospital, Aarhus, Denmark; 4 Department of Pharmacoepidemiology, Norwegian Institute of Public Health, Oslo, Norway; 5 Department of Epidemiology, Harvard School of Public Health, Boston, Massachusetts, United States of America; Istituto Superiore di Sanità, ITALY

## Abstract

**Background:**

The purpose was to describe utilization of selective serotonin reuptake inhibitors (SSRIs) and serotonin–norepinephrine reuptake inhibitors (SNRIs), including trends in prevalence, characteristics of users, drug switching and changes in prescribed doses in a large group of pregnant women across four Nordic countries.

**Methods:**

A drug utilization study based on linked individual-level data from the nationwide prescription- and medical birth registers in Denmark, Iceland, Norway and Sweden. The study population comprised all pregnancies in these countries, resulting in a live birth or stillbirth after gestational week 22 from January 1^st^ 2008 to December 31^st^ 2012 (N = 1 162 470). In addition to the main study drugs SSRIs and SNRIs, we included (concurrent) use of other antidepressants, antipsychotics, anxiolytics and hypnotics.

**Results:**

A total of 38 219 (3.3%) pregnancies were exposed to SSRIs and 5 634 (0.5%) to SNRIs. Prevalence of SSRI and SNRI use varied by country (1.8% in Norway to 7.0% in Iceland). Use and prescribed dosages decreased with each passing trimester of pregnancy; prevalence was 2.7% at conception, and 2.1%, 1.7% and 1.3% respectively in 1^st^, 2^nd^ and 3^rd^ trimester. In 0.6% of pregnancies women filled a prescription before pregnancy and in every trimester. In one third of exposed pregnancies, women were also dispensed anxiolytics, hypnotics or sedatives.

**Conclusion:**

Use of SSRI and SNRI use during pregnancy varied between the Nordic countries, but the overall prevalence remained low and relatively stable from 2008 to 2012. The low prevalence of use and high proportion of women who discontinue treatment in pregnancy raise questions about adequate treatment of depression in pregnant women.

## Introduction

Depression is estimated to occur in 7–15% of pregnancies in economically developed countries [1, 2]. Selective serotonin reuptake inhibitors (SSRIs) are the most frequently used antidepressants in pregnant women, with an estimated 2–3% of women in Europe and 4–10% in North America receiving such drugs during pregnancy [3–8]. In recent years serotonin-norepinephrine reuptake inhibitor (SNRIs) have increasingly been used as an alternative to SSRIs [9].

Decisions about using antidepressants during pregnancy are complex and require the weighing of several, often uncertain, factors, e.g. potential risks associated with exposure to antidepressant substances in-utero against risks of untreated depression. Based on available evidence, the European Medical Agency (EMA) and Food and Drug Administration (FDA) in the United States have issued warnings regarding use of SSRIs during late pregnancy and increased risk of persistent pulmonary hypertension in the newborn, as well as warnings of increased risk of congenital malformations, particularly cardiac defects, in association with exposure to paroxetine and fluoxetine during first trimester. Very recent epidemiological evidence suggests, however, that the excess risk for both persistent pulmonary hypertension [10] and birth defects [11, 12] may be more modest than previously indicated [13–16]. Current recommendations for treatment with SNRIs, such as venlafaxine and mirtazapine, during pregnancy are mainly based on earlier evidence for SSRIs rather than specific data on these newer substances.

Given the high prevalence of depression in women of childbearing age, promotion of optimal treatment during pregnancy is of major public health importance. Studies on antidepressant utilization during pregnancy have illustrated that a high proportion of women medicated for depression discontinue treatment once they become pregnant [3, 6, 17–19], often due to concerns of safety [20]. Yet, research related to the potential consequences of discontinued treatment, for mother and unborn infant, is quite scarce [21, 22]. Among women who do indeed continue treatment with SSRIs and SNRIs in pregnancy, more knowledge on potential changes in utilization patterns, e.g. drug switching and dosage changes, is needed to optimize treatment. Many, but not all [3], previous studies of the utilization and discontinuation of these antidepressants during pregnancy are based on self-reported surveys [4, 23, 24], insurance or reimbursement data [7, 6, 8, 17], or local pharmacy-dispensing data [25], potentially limiting the internal and external validity of the findings.

In this study, we sought to provide a recent and representative overview of how SSRIs and SNRIs are used among pregnant women under real life settings; identifying changes in utilization during pregnancy period, preferred substances, drug switching, dosages and psychotropic co-medication. Comparisons of drug use between geographical regions, demographic- and lifestyle factors may enhance health policies and practices leading to increased overall treatment success. Therefore, we further aimed to identify maternal characteristics related to use of these drugs, as well as variations by geography and secular time. To this end, we conducted a descriptive study of SSRI and SNRI utilization in a cohort of over 1.1 million pregnancies in four Nordic countries resulting in birth (after gestational week 22) between 2008 and 2012.

## Methods

### Study Setting

This was a descriptive observational drug utilization study based on nationwide data from prescription- and medical birth registers in four Nordic countries: Denmark, Iceland, Norway, and Sweden. The study population comprised all pregnancies in these countries, resulting in a live birth or stillbirth at gestational week 22 through 42 from January 1^st^ 2008 to December 31^st^ 2012 (N = 1 162 470).

Reporting to the nationwide health registers is mandatory in the Nordic countries and regulated by national laws. The national parliaments have on behalf of their populations given informed consent to be included in the registers [26]. The Civil Personal Registration number, a unique number assigned to each resident at birth or immigration, made it possible to merge information from the birth and prescription registers. From the birth registers we obtained information on maternal demographics and characteristics of pregnancy. The prescription registers of Norway, Sweden and Iceland cover reimbursed and non-reimbursed prescription drugs while the Danish National Database of Reimbursed Prescriptions only covers reimbursed prescription drugs including SSRIs and SNRIs. All registers include data on dispensed drug substance, brand name, and quantity (number of defined daily doses [DDDs]) together with dates of dispensing for over 95% of the total outpatient population [27]. From the Swedish register we also obtained prescription texts, dates of prescriptions and specialty department of prescribers. In general antidepressants are dispensed for a maximum of three months in the Nordic countries. The prescription registers do not hold complete information on the underlying indication for the drug treatment.

### Definitions of Drug Exposure during Pregnancy Period

We defined antidepressants according to the World Health Organization (WHO) Anatomical Therapeutic Chemical (ATC) classification [28] as substances within ATC-group N06A and focused on use of SSRIs (N06AB) and SNRIs (N06AX, venlafaxine and duloxetine) among pregnant women (S1 Table). The SNRIs milnacipran and desvenlafaxine were not used among pregnant women during the study years.

We estimated the **prevalence** of SSRI and SNRI use during pregnancy in each Nordic country, defined as the number of pregnant women per 100 pregnancies (%) in the study population, who filled at least one prescription for an SSRI or SNRI anytime from 90 days before the first day of last menstrual period (LMP) until delivery or end of pregnancy. Gestational length was based on the first day of LMP as estimated by prenatal ultrasound. We described drug use by trimester of pregnancy as a prescription fill during the following time windows: before pregnancy (up to 90 days before LMP), first trimester (0 to 97 days of gestation), second trimester (98 to 202 days of gestation) and third trimester (203 days of gestation to delivery). Use throughout whole pregnancy was defined as filling at least one SSRI/SNRI prescription in every pregnancy period.

Further, we compared women’s last SSRI/SNRI dispensing before LMP with their first SSRI/SNRI dispensing during gestational weeks 8 to 13 in effort to assess changing use in early pregnancy (once women become aware of their pregnancy); categorizing the latter prescription fill as for: the same drug, a new drug or no drug. We accounted for the preferred substances to which women switched by calculating the proportion (%) of pregnancies switching to that specific substance. In light of previous public health advisories regarding use of paroxetine in early pregnancy, we paid special attention to switches from paroxetine.

Among all SSRI/SNRI exposed pregnancies, we assessed concurrent use of tricyclic antidepressants (TCAs, N06AA), other antidepressants (N06AX, other than SNRIs), antipsychotics (N05A), anxiolytics (N05B), hypnotics and sedatives (N05C) by calculating the percentage of SSRI/SNRI exposed pregnancies in which a woman had, at anytime during pregnancy (-90 days < LMP ≤ delivery), also filled a prescription for a substance classified in the abovementioned groups. Information on use of antipsychotics (N05A) among pregnant women in Iceland and use of anxiolytics, hypnotics and sedatives (N05BA, N05CD) among pregnant women in Denmark was not available in the study data because these drugs are generally not reimbursed.

### Data Analysis

Using frequencies and proportions, as defined above, we described the prevalence and patterns of SSRI/SNRI use in pregnancy by drug group (SSRI, SNRI), specific substances (S1 Table), calendar year (defined by year of delivery), maternal country of residence (Denmark, Iceland, Norway, Sweden), age (≤24, 25–34, 35–44, ≥45 years), parity (0, 1, 2, 3, ≥4), relationship status (cohabiting with other parent, not cohabiting), smoking status during early pregnancy (smoker, non-smoker) and multiple pregnancy, i.e. pregnancies which led to birth of twins, triplets, etc. (yes, no).

In effort to describe SSRI/SNRIs dosage patterns during pregnancy, we analyzed prescription texts in the Swedish data and presented information on the prescribed dosage for each SSRI/SNRI by specialty department of the prescriber (general practice, obstetrics and gynecology, psychiatric, other) and trimester of pregnancy. The prescribed dosage was measured relative to the DDD, as the number of DDD units prescribed. Further, to assess changing dosages in early pregnancy we compared prescribed dosages from pregnancy weeks 8–13 with prescribed dosages from before LMP among women in Sweden who were dispensed SSRI/SNRIs in both periods. To assess changing dosages in late pregnancy we compared DDDs on the last prescription before delivery with women’s DDDs from before LMP. Prescription text data were inaccessible in Denmark, Iceland and Norway for this study.

We conducted an additional analysis to better capture changing use of SSRI/SNRIs in early pregnancy, by defining early pregnancy as gestational weeks 5–13, rather than 8–13 weeks, and compared women’s filled prescriptions from before LMP and in gestational weeks 5–13 with regard to specific SSRI/SNRIs and prescribed dosages.

All analyses were conducted using SAS software, version 9.4 (SAS Institute Inc., Cary, NC, USA). This study was approved by the regional ethical review board at the Karolinska Institutet in Sweden, the Danish Data Protection Agency and the National Board of Health; the National Bioethics Committee and the Data Protection Authority in Iceland and the Norwegian Data Inspectorate. The parliaments in the Nordic countries have on behalf of their populations given informed consent to be included in the national registers and the information recorded can be used for research purposes. Therefore, we did not obtain an informed written consent from women in the study population. All personal information was pseudonymized and de-identified prior to analysis.

## Results

Of 1 162 470 pregnancies in the population a total of 38 219 (3.3%) were exposed to SSRIs and 5 634 (0.5%) to SNRIs. Prevalence of SSRI/SNRI use varied slightly by age and parity, demonstrating u-shaped relations, with lowest use in women’s second pregnancy and ages 25–34 years. Prevalence was higher among smokers than non-smokers (7.9% vs. 3.2%), and among women not living with the other parent than those cohabitating (6.1% vs. 3.2%) (Table 1).

**Table 1:** Women exposed to SSRIs and SNRIs during pregnancy* by maternal characteristics, 2008–2012.

	*SSRI*		*SNRI*		*SSRI or SNRI*	
	*N*	Prev**	*N*	Prev**	*N*	Prev**
***Total***	38 219	3.3	5 634	0.5	42 178	3.6
***Country***						
*Denmark*	11 147	3.7	1 937	0.6	12 436	4.1
*Iceland*	1 634	7.0	211	0.9	1 753	7.5
*Norway*	5 417	1.8	857	0.3	6 128	2.0
*Sweden*	20 021	3.7	2 629	0.5	21 861	4.1
***Age*, *years***						
*-24*	6 301	3.7	883	0.5	6 905	4.1
*25–34*	22 643	3.0	3 339	0.4	24 981	3.4
*35–44*	9 202	3.7	1 399	0.6	10 213	4.1
*45+*	73	3.6	13	0.6	79	3.9
***Parity***						
*1*	12 058	3.2	1 704	0.5	13 303	3.5
*2*	8 663	2.8	1 037	0.3	9 401	3.0
*3*	4 267	3.5	633	0.5	4 726	3.9
*4+*	2 084	4.1	323	0.6	2 312	4.6
*Missing*	11 147	3.7	1 937	0.6	12 436	4.1
***Smoking status***						
*Non smoker*	27 992	2.9	3 800	0.4	30 691	3.2
*Smoker*	6 982	6.9	1 370	1.4	7 941	7.9
*Missing*	3 245	3.6	464	0.5	3 546	3.9
***Relationship status***						
*Cohabiting*	27 359	2.9	3 666	0.4	29 947	3.2
*Not cohabiting*	9 127	5.4	1 709	1.0	10 307	6.1
*Missing*	1 733	3.3	259	0.5	1 924	3.7
***Multiple pregnancies (twins*, *triplets*, *etc*.*)***						
*No*	37 622	3.3	5 523	0.5	41 492	3.6
*Yes*	597	3.0	111	0.6	686	3.5

* including 90 days period before LMP

** prevalence per 100 women

**Fig 1:**
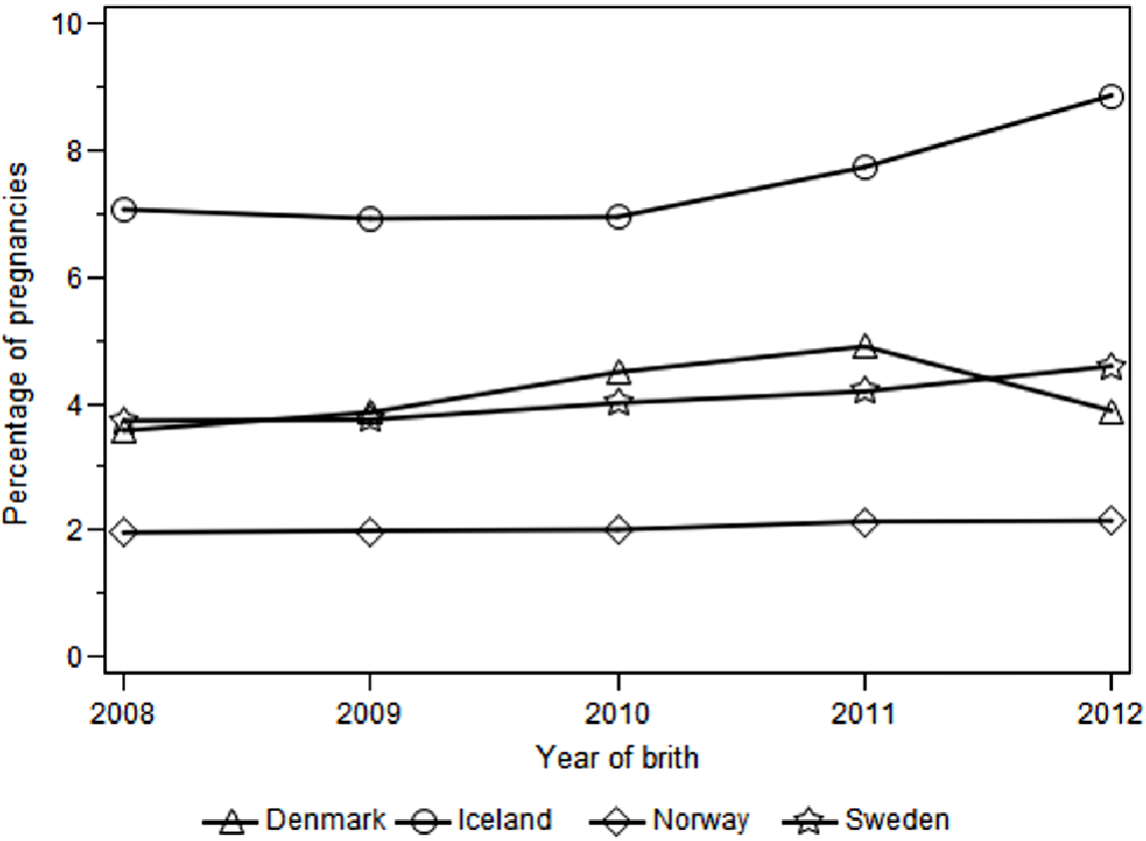
Prevalence* of SSRI/SNRI use per 100 pregnancies by year of delivery and country of residence. * at least one dispensed SSRI/SNRI during pregnancy, including 90 days period before LMP

Prevalence of SSRI/SNRI use in pregnancy remained relatively stable from 2008 to 2012 but the overall estimate for 2008–2012 varied by country; ranging from 1.8% in Norway, 3.7% in Denmark and Sweden, to 7.0% in Iceland (Fig 1. Prevalence* of SSRI/SNRI use per 100 pregnancies by year of delivery and country of residence).

**Fig 2:**
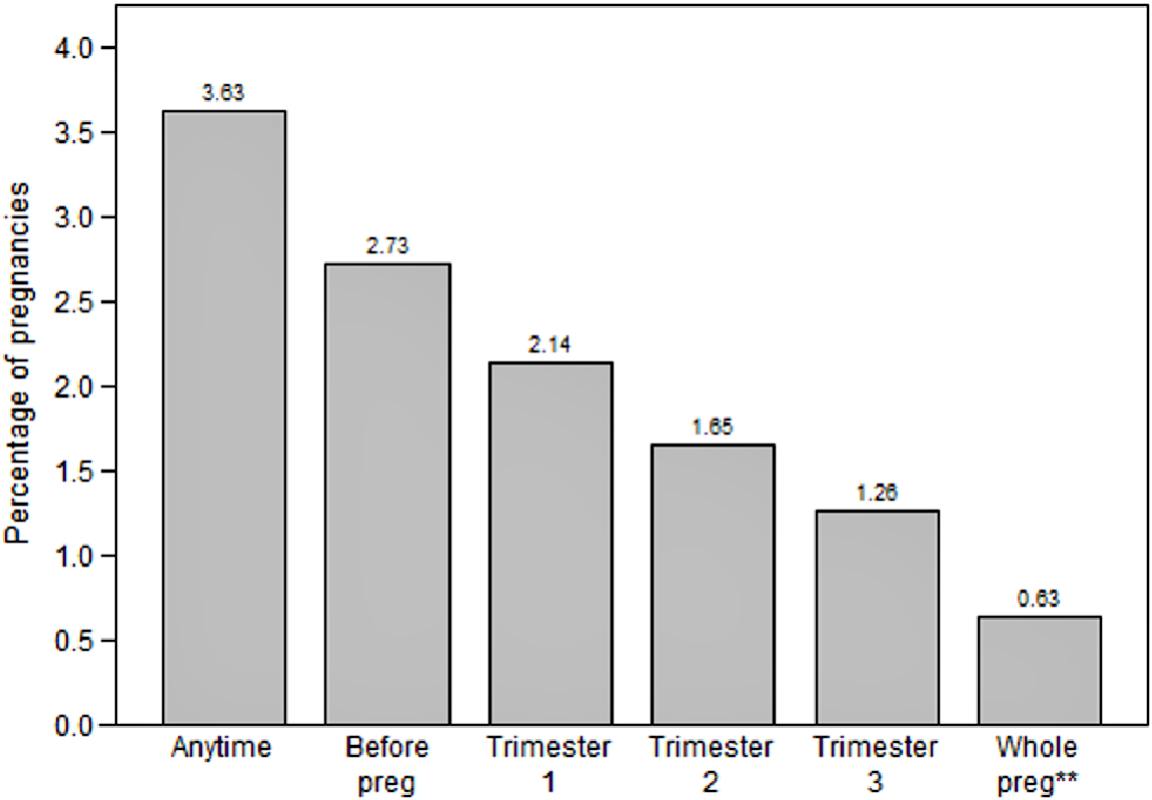
Prevalence of SSRI/SNRI use per 100 pregnancies in the Nordic population by trimester. Preg, pregnancy * at least one dispensed SSRI/SNRI during relevant time window per 100 pregnancies ** whole pregnancy = at least one dispensed SSRI or SNRI during each trimester of pregnancy, including 90 days period before LMP

Use was most prevalent before LMP (2.7%) and decreased with each passing trimester, with 0.6% of pregnancies exposed throughout the whole pregnancy period, i.e. with at least one SSRI/SNRI dispensing within 90 days before LMP and one dispensing in every trimester (Fig 2. Prevalence of SSRI/SNRI use per 100 pregnancies in the Nordic population by trimester).

**Fig 3:**
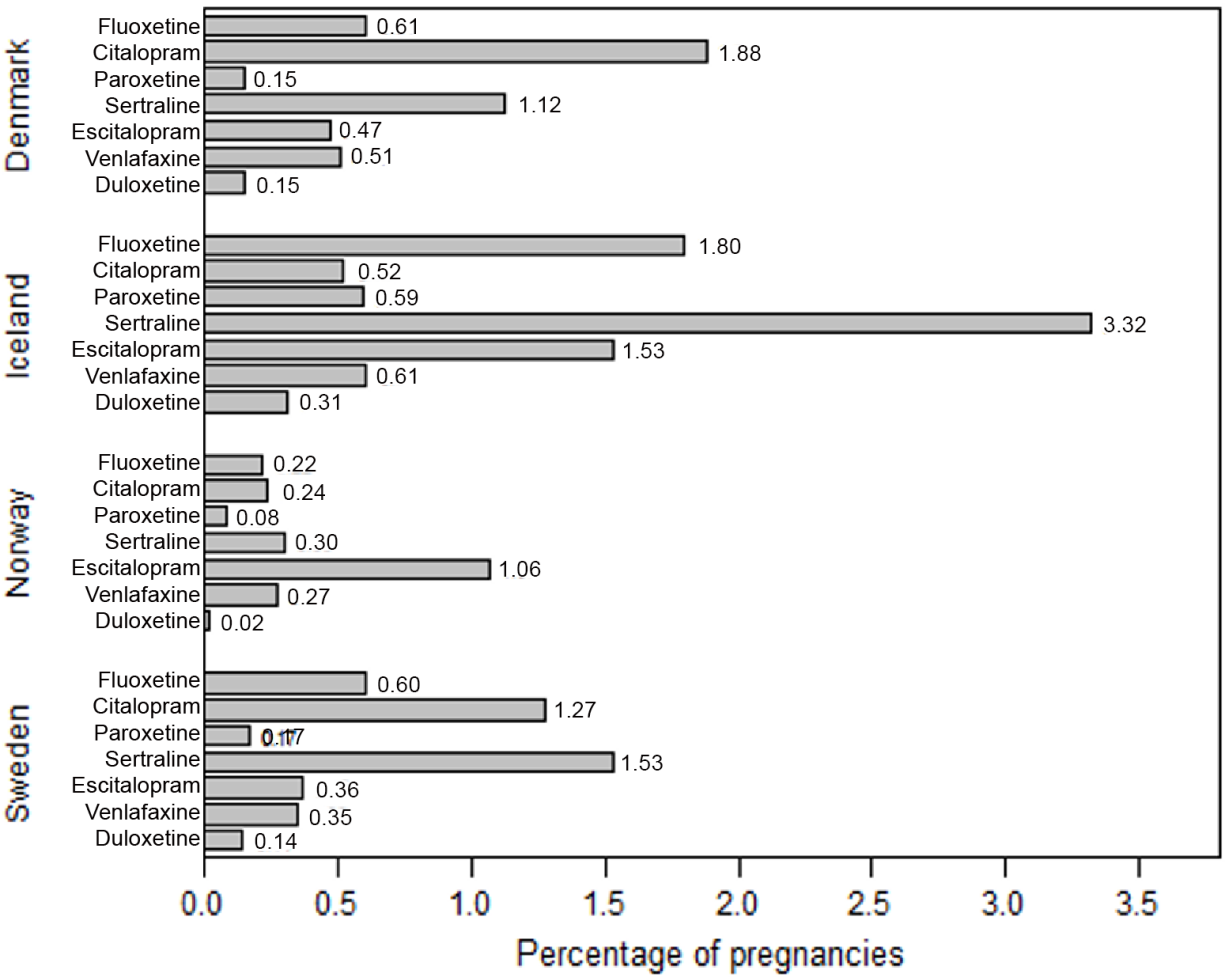
Prevalence* of most commonly used SSRI/SNRI substances per 100 pregnancies by country. * at least one dispensed SSRI/SNRI during pregnancy, including 90 days period before LMP.

The most commonly used substances during pregnancy differed somewhat by country but were mainly sertraline, citalopram, escitalopram and fluoxetine (Fig 3. Prevalence* of most commonly used SSRI/SNRI substances per 100 pregnancies by country).

Among 18 129 pregnancies in which women filled an SSRI/SNRI prescription before LMP, 21.4% were dispensed the same drug in gestational weeks 8–13; 1.8% switched to another SSRI or SNRI substance; but 76.8% did not fill another prescription for such drugs in early pregnancy (Table 2). The proportion not filling another prescription in early pregnancy varied somewhat by country (73.2% in Demark, 79.7% in Iceland, 85.4% in Norway, and 75.6% in Sweden).

**Table 2:** Last SSRI/SNRI dispensing before LMP* and first dispensing in early pregnancy (weeks 8–13).

*Before LMP*		*Week 8–13*					
		*Same drug*		*New drug*		*No drug*	
	*N*	*N*	*%*	*N*	*%*	*N*	*%*
*Fluoxetine*	1 996	467	23.4	17	0.9	1 512	75.8
*Citalopram*	5 598	1 258	22.5	74	1.3	4 266	76.2
*Paroxetine*	731	105	14.4	54	7.4	572	78.2
*Sertraline*	4 701	1 165	24.8	25	0.5	3 511	74.7
*Fluvoxamine*	12	3	25.0	.		9	75.0
*Escitalopram*	3 062	453	14.8	66	2.2	2 543	83.1
*Venlafaxine*	1 552	378	24.4	64	4.1	1 110	71.5
*Duloxetine*	477	54	11.3	25	5.2	398	83.4
*Total*	18 129	3 883	21.4	325	1.8	13 921	76.8

* within 90 days before last menstrual period

Among the 325 pregnancies in which women switched to another substance in early pregnancy, the preferred new antidepressant was sertraline (40.6%) followed by citalopram (22.5%) and escitalopram (5.8%). Most switches occurred in pregnancies where women had been exposed to paroxetine before LMP (7.4%, 54 of 731).

Table 3 demonstrates that the mean prescribed dosage of SSRI/SNRIs decreased slightly during the course of pregnancy among women in Sweden, from 1.36 DDD before LMP to 1.23 DDD in third trimester. On average, specialists within psychiatry prescribed the highest SSRI/SNRIs dosages and specialist in obstetrics and gynecology prescribed the lowest dosages.

**Table 3:** Number of filled prescriptions (N) and prescribed defined daily doses (DDD) during pregnancy according to text analysis of Swedish prescriptions of SSRI/SNRIs.

		*Prescribed DDD*		
	*N*	*Mean*	*Q1*	*Q3*
***Total***	40 758	1.32	1.00	1.50
***Prescriber´s department***				
*General Practice*	19 636	1.22	1.00	1.50
*Obstetrics & Gynecology*	1 635	1.06	1.00	1.00
*Psychiatric*	16 215	1.47	1.00	2.00
*Other*	3 169	1.31	1.00	1.50
*Unknown*	103	1.22	1.00	1.50
***Time of exposure***				
*Before LMP*	22 147	1.36	1.00	2.00
*Trimester 1*	8 478	1.28	1.00	1.50
*Trimester 2*	6 721	1.27	1.00	1.50
*Trimester 3*	3 412	1.23	1.00	1.50

Q, quartile; LMP, last menstrual period

Restricting the analysis of prescribed dosage to pregnancies exposed both before LMP and in early pregnancy (weeks 8–13), the mean prescribed dosage decreased by 0.17 (from 1.43 DDD to 1.27 DDD), with the number of DDD unchanged, decreased or increased, respectively, in 53.8%, 31.2% and 15.0% of instances. Restricting the analysis to pregnancies exposed both before LMP and in third trimester, the mean prescribed dosage decreased by 0.15 (from 1.45 DDD before LMP to 1.29 DDD on last prescription in third trimester), with the number of DDD unchanged, decreased or increased, respectively, in 49.6%, 32.6% and 17.7% of instances.

Of all SSRI/SNRIs exposed pregnancies 8 727 (29.2%) were also exposed to anxiolytics, hypnotics or sedatives, 2 186 (5.4%) to antipsychotics and 2 636 (6.2%) to other antidepressants. As with prevalence of SSRI/SNRI use, concurrent drug use showed a u-shaped association with age and it was elevated among smokers (41.1%, 9.7% and 8.7%, respectively for each aforementioned drug group) and non-cohabitating women (40.3%, 8.1% and 7.4% respectively for each aforementioned drug group).

The changes in patterns of SSRI/SNRI use and dosages from before LMP to early pregnancy remained nearly the same in an additional analysis, where early pregnancy was defined as pregnancy weeks 5–13, as in the main analysis.

## Discussion

In this large population-based study covering over 1.1 million pregnancies, carried to at least gestational week 22, in four Nordic countries, we found an overall 3.6% prevalence of SSRI and SNRI use among pregnant women, varying over 3-fold between countries. Use of SSRIs and SNRIs decreased with each passing trimester of pregnancy; less than one fourth of women medicated at conception continued this treatment into pregnancy. In one third of exposed pregnancies, women were also dispensed anxiolytics, hypnotics or sedatives while pregnant. Sertraline, citalopram, escitalopram and fluoxetine were the most frequently used substances, but to a differing degree by country.

This is the first study to comprehensively describe changing patterns of SSRIs and SNRIs utilization among pregnant women across Nordic countries. Jimenez-Solem et al. [3] recently provided a national overview of antidepressant use among pregnant women in Denmark, showing a 16-fold increase in exposure to any antidepressant between 1997 and 2010 (from 0.2% to 3.2%). Our findings, however, indicate a rather stable prevalence of SSRI/SNRI use between 2008 and 2012 in Denmark, Norway and Sweden, but increasing use among pregnant women in Iceland.

The prevalence of SSRI/SNRI use had a u-shaped association with both age and parity, as use dropped slightly among women aged 25–34 years and in second pregnancy in our data. Earlier studies have, on the other hand, generally shown higher antidepressant use in older than younger pregnant women [6, 29, 5, 8]. As expected [3, 6], we found that pregnant women who smoked were more likely than non-smokers to use SSRIs or SNRIs (7.9% vs. 3.2%), similarly women who did not cohabitate with their partner were more likely, than cohabitating women, to be exposed to SSRI/SNRIs with (6.1% vs. 3.2%). Knowledge of such demographic and lifestyle characteristics may be informative for policy-makers and health-care workers managing pharmacologic treatment of depression in pregnant women. Previous research from Denmark suggests that women of childbearing age with an unhealthy lifestyle are at least 1.5-fold more likely to use SSRIs than those with a healthy lifestyle [30], suggesting the importance of monitoring for lifestyle factors as well as underlying depression during prenatal care.

The observed between-country variation in SSRI/SNRIs use (1.8% to 7.0%) is in line with Huybrechts et al. [6] findings on publicly insured women in the United States 2000–2007, where the proportion of pregnant women exposed to antidepressants ranged from 2.7% (Hawaii) to 22.6% (Maine). Even after accounting for various potential between-state differences (e.g. patient demographics, case mix and calendar year), a 4-5-fold difference in use between states remained [6]. Likewise, Charlton et al. recently showed differences in prevalence of SSRI prescribing to pregnant women between six European regions, ranging from 1.2% to 4.5% [19]. The generally widespread use of antidepressants and other psychotropic drugs in Iceland has been previously described, in studies [31] [32] and reports [33]. Reasons for differential prevalence of SSRI/SNRI use within the relatively homogeneous Nordic populations are likely to lie in physicians’ prescribing culture, access and reimbursement of non-pharmacological treatment options, rather than different underlying disease rates.

We note that the overall proportion of women exposed to SSRI/SNRIs in Denmark, Iceland Norway and Sweden in 2008–2012 did not seem to exceed the estimated 7–15% prevalence of depression during pregnancy [1, 2]. But drug treatment prevalence, per se, is not sufficient to conclude on appropriateness of drug use during pregnancy. Aside from depression, SSRIs and SNRIs can be prescribed for a wide range of conditions to pregnant women, e.g. generalized anxiety disorder, panic attacks, pain, chronic fatigue syndrome, smoking problems, etc. [6] and we did not have access to information on the underlying indication for which drugs were prescribed. To better assess SSRI/SNRI utilization during the pregnancy period we therefore examined potential changes in treatment, especially in early and late pregnancy.

In our data switching of substances in early pregnancy was most common in women exposed to paroxetine, fitting with earlier public health warnings of an increased risk of congenital malformations associated with use of paroxetine in first trimester, as well as with the summary of product characteristics (SPC) for paroxetine. Although the Nordic countries do not have common clinical guidelines for treating depression in pregnant women, our finding, that sertraline was the preferred drug of choice for women switching to a new substance, is in line with clinical guidelines from Denmark recommending sertraline or citalopram as first-line drugs when starting treatment in pregnant women. While our analyses indicated diminishing prescribed SSRI/SNRI dosages by trimester of pregnancy, evidence does not necessarily suggest tapering or stopping SSRI treatment towards the end of pregnancy to be associated with improved neonatal outcomes [34]. On the contrary, dose increases may be indicated for many SSRIs, especially late in pregnancy, due to the pharmacokinetic changes that can occur during pregnancy [35].

Conforming to a pattern demonstrated in previous studies [8, 6, 3, 36], most women in our data discontinued SSRI/SNRI use in early pregnancy. This tendency, which is most likely due to earlier concerns of harmful drug effects, e.g. risk of birth defects [12, 11, 10, 37] or obstetrical and perinatal complications [38], brings forth concerns of possible under-treatment of depression among pregnant women. In fact, some, yet not all [39], systematic evidence suggests that the prevalence of maternal depression is elevated in second and third trimesters [2]. Based on monthly psychiatric assessments of 201 pregnant women with a history of major depression, Cohen et al. [21] showed that women who discontinued antidepressant treatment close to conception were more likely to relapse later in pregnancy, than those who maintained antidepressant treatment. Recent evidence suggesting low, if any, risks of perinatal death [40] or birth defects [11, 12] by SSRI use during pregnancy. Also, untreated maternal depression may confer multiple health risks for both mother and unborn child [41, 38], both of which calls for reconsideration of policy on pharmaceutical treatment of maternal depression during pregnancy. Optimal treatment approaches should be evaluated continuously through pregnancy in women with depressive symptoms, irrespective of what risk-benefit decision was taken at the beginning of pregnancy.

### Strengths and Limitations

Our study covers an entire population of pregnant women in four countries, who gave birth after gestational week 22, and it is based on detailed data from the nationwide medical birth- and prescription registers, linked via unique personal identification numbers. The study has several limitations. Firstly, it does not include information on SSRI/SNRI use in pregnancies terminated before gestational week 22. Since we have previously shown that SSRI users are more likely than non-users to have an elective termination of pregnancy at 12–23 weeks [42], the prevalence of SSRI use among all pregnant women is likely to be somewhat higher than demonstrated here. Secondly, we did not have information on the indication for drug use and could only assess prescribed dosages from Sweden. Therefore, we are unable to firmly conclude on the appropriateness of SSRI/SNRI prescribing to pregnant women in the population. Thirdly, we did not know whether women had already used antidepressants before pregnancy, (i.e. over three months before becoming pregnant) or whether they started treatment during pregnancy, as the study data only covered a three-month period prior to women’s LMP. Therefore, the study is not informative regarding the initiation of SSRI/SNRI treatment during pregnancy. Fourthly, we did not have access to data on antipsychotic use in Iceland or non-reimbursable benzodiazepines in Denmark, thus the prevalence concurrent use of these drugs with SSRI/SNRIs could not be estimated for these two countries. Finally, as in most registry-based studies we did not know whether, or during which trimester, women actually consumed the dispensed drugs. In this descriptive study we considered information on single, as well as multiple, prescription fills during pregnancy of interest to describe patterns of use.

## Conclusions

In sum, this study demonstrates rather low, yet somewhat differing, use of SSRIs and SNRIs among pregnant women across four Nordic countries. The prevalence of SSRI and SNRI use decreases during pregnancy but it remains unknown whether this is a signal of inadequate treatment of depression in pregnant women.

## Supporting Information

S1 Table Categorization of antidepressants with defined daily doses (DDDs) and frequency of prescription fills (N) for each substance. SSRIs, selective serotonin reuptake inhibitors; SNRIs, serotonin–norepinephrine reuptake inhibitors.
(DOCX)
