# Supplementary material for: Use of SSRI and SNRI Antidepressants during Pregnancy: A Population-Based Study from Denmark, Iceland, Norway and Sweden
Source: PLoS One. 2015 Dec 14;10(12):e0144474. doi: 10.1371/journal.pone.0144474 (PMC4685993; doi:10.1371/journal.pone.0144474)
Supplement: S1 Table — SSRIs, selective serotonin reuptake inhibitors; SNRIs, serotonin–norepinephrine reuptake inhibitors. (DOCX) [file pone.0144474.s001.docx]

| **S1 Table.** Categorization of antidepressants with defined daily doses (DDDs) and frequency of prescription fills (N) for each substance. | | | | | |
| --- | --- | --- | --- | --- | --- |
| *Group* | *ATC code* | *Drug name* | *DDD* | *N* | *Percent of Group Frequency* |
| SSRI | N06AB03 | Fluoxetine | 20 mg | 18 456 | 16.5 |
|  | N06AB04 | Citalopram | 20 mg | 34 954 | 31.3 |
|  | N06AB05 | Paroxetine | 20 mg | 4 139 | 3.7 |
|  | N06AB06 | Sertraline | 50 mg | 37 079 | 33.2 |
|  | N06AB08 | Fluvoxamine | 0.1 g | 103 | 0.09 |
|  | N06AB10 | Escitalopram | 10 mg | 16 942 | 15.2 |
| SNRI | N06AX16 | Venlafaxine | 0.1 g | 15 801 | 79.3 |
|  | N06AX21 | Duloxetine | 60 mg | 4 122 | 20.7 |
| SSRIs, selective serotonin reuptake inhibitors  SNRIs, serotonin–norepinephrine reuptake inhibitors | | | | | |
